# Palladium Catalyzed Heck Arylation of 2,3-Dihydrofuran—Effect of the Palladium Precursor

**DOI:** 10.3390/molecules19068402

**Published:** 2014-06-19

**Authors:** Adam Morel, Anna M. Trzeciak, Juliusz Pernak

**Affiliations:** 1 Faculty of Chemistry, University of Wrocław, 14 F. Joliot-Curie St., 50-383 Wrocław, Poland; 2 Department of Chemical Technology, Poznań University of Technology, pl. M. Skłodowskiej-Curie 2, 60-965 Poznań, Poland

**Keywords:** palladium, Heck coupling, ionic liquids, nanoparticles

## Abstract

Heck arylation of 2,3-dihydrofuran with iodobenzene was carried out in systems consisting of different palladium precursors (Pd_2_(dba)_3_, Pd(acac)_2_, PdCl_2_(cod), [PdCl(allyl)]_2_, PdCl_2_(PhCN)_2_, PdCl_2_(PPh_3_)_2_) and ionic liquids (CILs) with L-prolinate or L-lactate anions. All the tested CILs caused remarkable increases of the conversion values and in all of the reactions 2-phenyl-2,3-dihydrofuran (**3**) was obtained as the main product with a yield of up to 59.2%. The highest conversions of iodobenzene were achieved for the [PdCl(allyl)]_2_ precursor. Formation of Pd(0) nanoparticles, representing the resting state of the catalyst, was evidenced by TEM.

## 1. Introduction

**Scheme 1:**
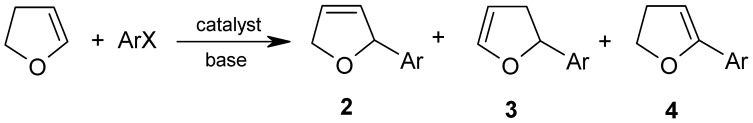
Heck arylation of 2,3-dihydrofuran.

The Heck reaction is one of the most important catalytic organic reactions leading to arylated olefins [1,2,3,4,5,6,7]. Application of a cyclic olefin as a substrate of the Heck reaction enables one to perform it in an asymmetric way [8,9,10,11,12,13]. In this context, arylation of DHF is often explored as a model reaction to study regioselectivity and enantioselectivity in the presence of different palladium catalysts, mainly containing chiral phosphorus ligands [9,10,11,12,13,14,15,16,17,18,19,20,21,22,23,24]. Arylation of DHF takes place exclusively in C2 position, however, as a consequence of double bond migration, two other products might also be formed (Scheme 1). Product **2** is the kinetic one, whereas **3** is thermodynamic [25,26,27,28].

We have shown for the first time that product **3** can be obtained with the excellent enantioselectivity in a phosphine-free system with Pd(OAc)_2_ as the catalyst precursor and [NBu_4_][L-PRO] as the chiral agent [22]. For that system we proposed the homogeneous halide-free pathway as a dominant in the selective formation of arylated dihydrofurans.

In order to learn more about the mechanism of DHF arylation we concentrated our efforts on evaluation of different palladium precursors to compare their applicability in place of Pd(OAc)_2_. We selected five phosphine-free palladium complexes, Pd_2_(dba)_3_, Pd(acac)_2_, PdCl_2_(cod), [PdCl(allyl)]_2_, PdCl_2_(PhCN)_2_ and PdCl_2_(PPh_3_)_2_. On the basis of the previous results, four CILs were chosen, three containing L-prolinate anion and one L-lactate.

Despite of chirality of CILs, preliminary catalytic tests shown low enantioselectivity of the studied reactions. Therefore we focused our investigations on the selection of the best palladium precursor facilitating high conversion of substrates. It is well accepted in the literature that under catalytic reaction conditions palladium precursors are transformed to catalytically active forms, monomolecular complexes or palladium nanoparticles [29,30,31,32,33,34,35,36,37,38,39]. In this context it was interesting to study relation between structure of palladium precursor and its reactivity in the Heck reaction.

## 2. Results and Discussion

### 2.1. Arylation of DHF at the Presence of [DDA][L-PRO]

**Figure 1:**
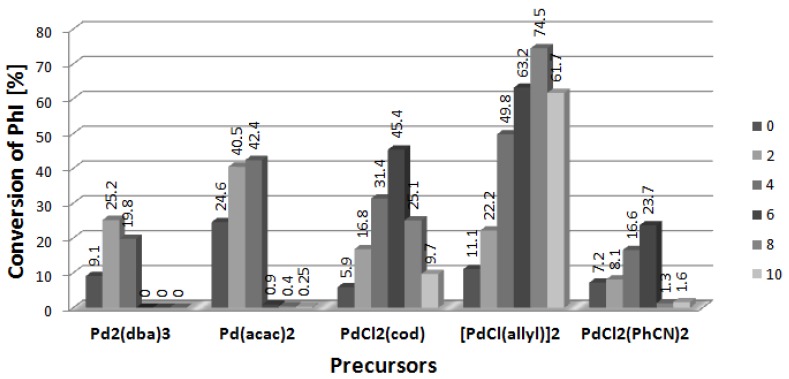
Effect of [CIL]:[Pd] ratio on conversion of PhI in Heck arylation of DHF. CIL = [DDA][L-PRO].

Figure 1 presents results obtained in the presence of [DDA][L-PRO] (DDA = didodecyldimethyl-ammonium cation, L-PRO = L-prolinate) used in 2–10-fold excess to palladium which was applied in amount of 1% mol. In all the cases, the conversion of iodobenzene was higher after addition of two equivalents of CIL than in the reference reaction carried out with palladium complexes only. When the amount of CIL exceeded 2, conversion continued to increase, however, that positive effect was observed only to [CIL]/[Pd] equal 4–6, and then the conversion dropped down. In some systems the reaction was stopped completely at [CIL]/[Pd] = 10. The best results, up to 74.5% of the arylated products, were obtained for dimeric complex [PdCl(allyl)]_2_. Interestingly, relatively low conversions were noted for Pd_2_(dba)_3_, the only Pd(0) species in the studied series. Reactivity of PdCl_2_(PPh_3_)_2_ differs significantly from that of the phosphine-free palladium precursors and already a 2-fold amount of CIL inhibited the reaction totally. PdCl_2_(PPh_3_)_2_ used without addition of [DDA][L-PRO] enabled to obtain 38.5% conversion to arylated products, mainly product **3** (34.6%).

**Figure 2:**
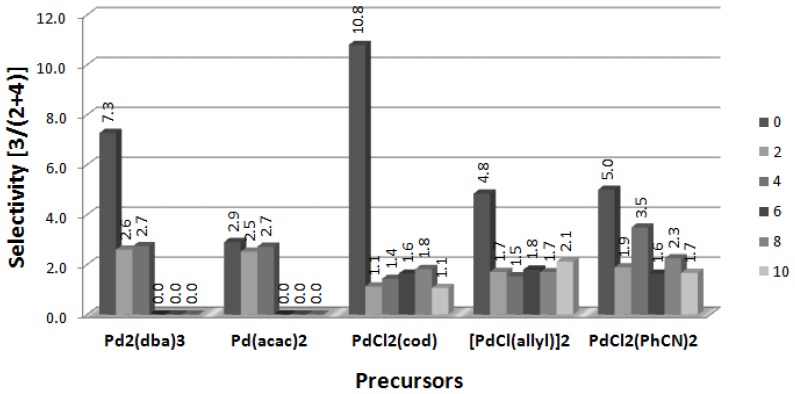
Effect of [CIL]:[Pd] ration on selectivity of Heck arylation of DHF. CIL = [DDA][L-PRO].

Data collected in Figure 2 illustrate the changes of the Heck reaction selectivity at the presence of [DDA][L-PRO], expressed as the ratio [**3**/(**2** + **4**)]. It is clear that addition of CIL caused a decrease of selectivity towards product **3**, which was the main one in all the reactions performed in unmodified palladium systems. The biggest decrease of selectivity, caused by the presence of CIL, was noted for PdCl_2_(cod). It can be concluded that [DDA][L-PRO] diminished isomerization of the double bond responsible for transformation of **2** to **3**. Exceptionally, such an effect was practically not observed in the reactions with Pd(acac)_2_, in which the selectivity of the reactions performed with and without CIL was almost the same.

### 2.2. Arylation of DHF at the Presence of [BA][L-PRO]

**Figure 3:**
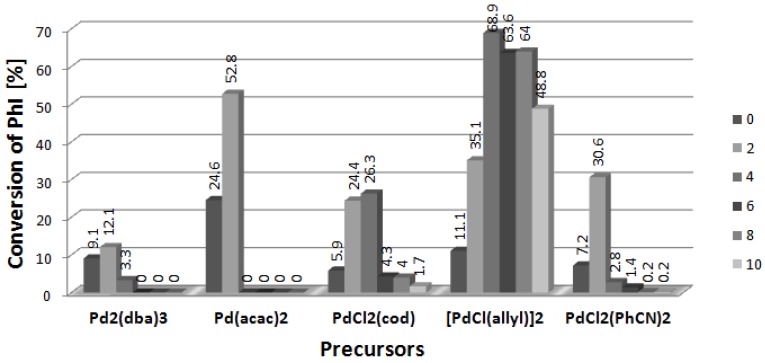
Effect of [CIL]:[Pd] ratio on conversion of PhI in Heck arylation of DHF. CIL = [BA][L-PRO].

The influence of [BA][L-PRO] (BA = cation with C_12_H_25_ and C_14_H_29_ alkyl groups in proportions equal to 60% and 40% respectively) on the Heck arylation of DHF is shown in Figure 3. Similarly as for [DDA][L-PRO], all the tested palladium precursors showed the increase of catalytic activity at the presence of CIL. Moreover, the inhibiting effect at higher concentrations of [BA][L-PRO] was even stronger than that of [DDA][L-PRO] and in most cases at the 4-fold excess of CIL only traces of the products were obtained. In fact, the studied systems are applicable only with a 2-fold excess of [BA][L-PRO]. The only different dependence was found for [PdCl(allyl)]_2_ which achieved reasonable conversions in the whole range of [Pd]/[CIL] from 2 to 10, with the maximum (68.9%) at the 4-fold excess of CIL.

The presence of [BA][L-PRO] in the catalytic reaction also influenced the selectivity towards **3** and generally caused its decrease. Such an effect is particularly noticeable for the PdCl_2_(cod), forming dominantly product **3** (**3**/(**2**+**4**) = 10.8). After addition of CIL, the yield of **2** and **4** increased and as a result the parameter **3**/(**2**+**4**) decreased to 1.2–2.9. It is also worth to note that changes of [BA][L-PRO] concentration have relatively small influence on the reaction selectivity.

### 2.3. Arylation of DHF at the Presence of [NBu_4_][L-PRO]

**Figure 4:**
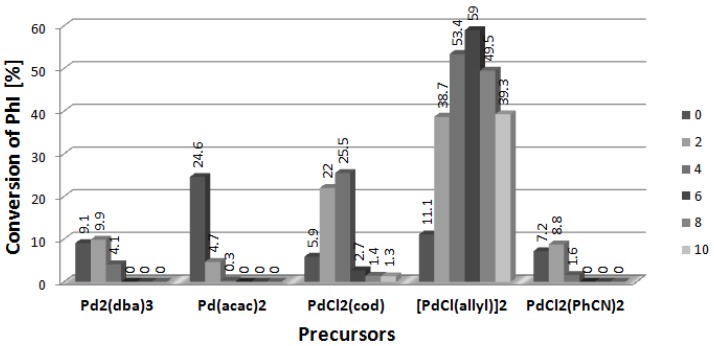
Effect of [CIL]:[Pd] ratio on conversion of PhI in Heck arylation of DHF. CIL = [Bu_4_N][L-PRO].

The results of testing of the next CIL with the same anion, [NBu_4_][L-PRO], are presented in Figure 4. The best results were obtained again for the dimeric [PdCl(allyl)]_2_, which produced 59% of arylated products at the [CIL]/[Pd] ratio 6. Further increase of the amount of [NBu_4_][L-PRO] caused a decrease of iodobenzene conversion to 39.3% at [CIL]/[Pd] = 10. It was also possible to get *ca.* 25% of the products with application of PdCl_2_(cod). Here the ee value estimated for product **2** reached 19.7, whereas for **3** it was close to 6. Other palladium precursors presented relatively low productivity when used with [NBu_4_][L-PRO].

Similarly as for the previously studied L-prolinate salts, the biggest change of the **3**/(**2**+**4**) parameter caused by the presence of [NBu_4_][L-PRO] was observed for PdCl_2_(cod). Interestingly, the selectivity of the Heck coupling with the most efficient complex, [PdCl(allyl)]_2_, was not very sensitive to CIL which caused a change of the **3**/(**2**+**4**) value from 4.8 to 2.3–2.5.

### 2.4. Arylation of DHF at the Presence of [NBu_4_][L-LAC]

**Figure 5:**
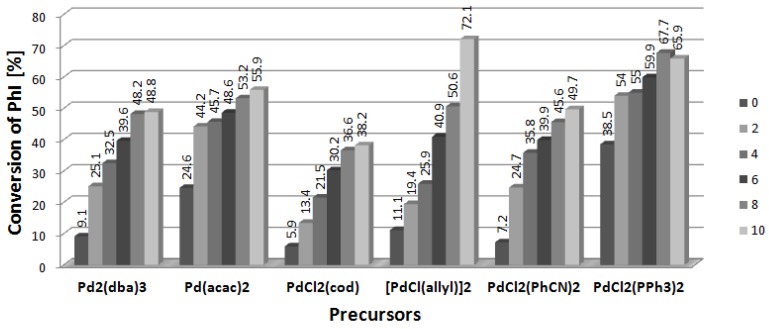
Effect of [CIL]:[Pd] ratio on conversion of PhI in Heck arylation of DHF. CIL = [Bu_4_N][L-LAC].

The highest conversions of iodobenzene for all palladium precursors were found for [NBu_4_][L-LAC] (L-LAC = L-lactate anion), applied as 70% solution in water (Figure 5). The presence of CIL caused the increase of the yield of the arylated dihydrofurans in comparison to the reactions performed without additives, similarly as it was observed in the previous systems. However, in contrast to L-prolinate salts, [NBu_4_][L-LAC] did not cause any inhibiting effect and the increase of its concentration resulted in a systematic increase of iodobenzene conversion (Figure 5). Interestingly, Pd_2_(dba)_3_ complex was also activated with [NBu_4_][L-LAC] and formed up to 48.8% of the products. When Pd(acac)_2_ was used as the catalyst precursor, the conversion increased from 24.6% to 44.2% at the 2-fold excess of CIL and further improved to 55.9% when [CIL]/[Pd] was increased to 10. At the same time the ee value increased from 0.2 to 11.8. Thus, the effect of CIL on enantioselectivity was quite remarkable in this case. Dimer [PdCl(allyl)]_2_ exhibited a linear increase of iodobenzene conversion with the rise of [NBu_4_][L-LAC] amount and the best result, 72.1%, was obtained when [CIL]/[Pd] was 10. However, the ee values remained below 5. The next two palladium precursors, PdCl_2_(PhCN)_2_ and PdCl_2_(PPh_3_)_2_, were nicely activated by [NBu_4_][L-LAC], giving products with the yields of 49.7% and 67.7%, respectively. Asymmetric induction was also noted, in particular for product **3**, however the ee values did not exceed 6.6.

At the presence of [NBu_4_][L-LAC] the values of **3**/(**2**+**4**) were higher for all the precursors. Interestingly, in reactions with PdCl_2_(PPh_3_)_2_ precursor, relatively high values of the selectivity parameter **3**/(**2**+**4**), *ca.* 7, were noted. At the same time the total yield of the products exceeded 60%, *ca.* 50%–59% of **3** was formed together with *ca.* 6.5% of **2**. Considering the regioselectivity, the catalytic system containing PdCl_2_(PPh_3_)_2_ and [NBu_4_][L-LAC] is superior over all other studied systems.

It was suspected that the very positive effect of [NBu_4_][L-LAC] on the catalytic activity of palladium precursors could be explained by an influence of water, the component of the CIL solution. To check that possibility, experiments were carried out with the application of Pd(acac)_2_, [BA][L-PRO] and 30–90 µL of water. The obtained results did not show any influence of water on the reaction course.

### 2.5. Arylation of DHF with [PdCl(allyl)]_2_ Precursor

**Figure 6:**
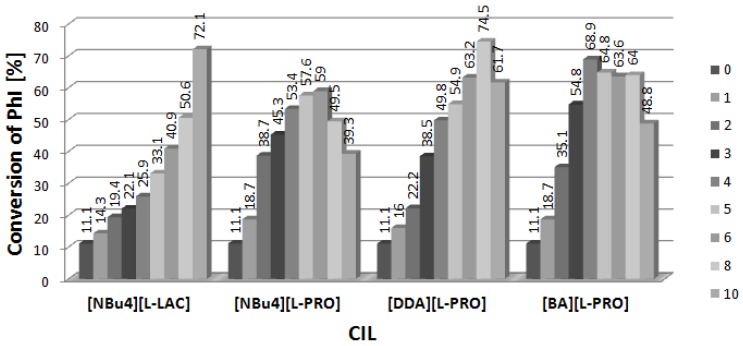
Heck arylation of DHF catalyzed by [PdCl(allyl)]_2_ and CILs.

The performed studies enabled to select the most efficient catalytic systems for the Heck arylation of DHF. Considering the conversion of iodobenzene, the best results were obtained with [PdCl(allyl)]_2_ precursor which formed very efficient systems with all the CILs used. The conversion of iodobenzene reached maximum 74.5% and two CILs, namely [NBu_4_][L-LAC] and [DDA][L-PRO] showed the best results (Figure 6).

Interestingly, [PdCl(allyl)]_2_ was also indicated as superior palladium precursor in Heck cross-coupling between 4-nitrochlorobenzene and styrene [29].

In order to get deeper knowledge about transformations of [PdCl(allyl)]_2_ under catalytic reaction conditions, spectroscopic studies were undertaken. First, coordination of L-prolinate anion to palladium was considered.

It was expected that eventual coordination of L-prolinate to palladium should be visible in ^1^H-NMR. To estimate such effect ^1^H-NMR spectra of L-proline and Pd(L-PRO)_2_ complex [40] were analyzed (Table 1) Difference of chemical shift of CH protons in free and coordinated proline was equal 0.14 ppm. Moreover, in free L-proline signals CH_2_-3 and CH_2_-4 appeared as two multiplets each while only one signal of CH_2_-3 protons was observed in the spectrum of Pd(L-PRO)_2_. In general, coordination of L-proline to palladium caused the upfield shift of all signals in ^1^H-NMR spectrum. Next, spectra of [BA][L-PRO] and a sample containing [BA][L-PRO] and [PdCl(allyl)]_2_ were analyzed. In both spectra chemical shifts were similar, the only difference was overlapping of signals originated from CH_2_-3 which presented two multiplets in the spectrum of [BA][L-PRO]. Such effect can indicate on week interaction of L-proline anion with palladium rather than on the formation of coordinated compound.

**Table 1:** ^1^H-NMR data of L-proline (**a**) and Pd(L-PRO)_2_ (**b**) in D_2_O; [BA][L-PRO] and [BA][L-PRO]/[PdCl(allyl)]_2_ = 1 in CDCl_3_.

(a)			(b)		
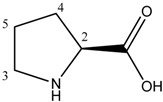			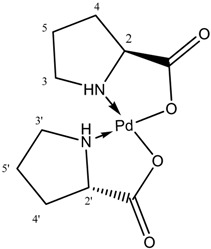		
	**L-proline**	**Pd(L-PRO)_2_**	**[BA][L-PRO]**	**[BA][L-PRO]/[PdCl(allyl)]_2_**
Proton	δ (ppm)	δ (ppm)	δ (ppm)	δ (ppm)
CH-2	4.04 t (*J* = 7.8 Hz)	3.90 t (*J* = 8.4 Hz)	3.70 m	3.71 m
CH_2_-3a	3.32 m	3.14 m	3.10 m	2.19 m
CH_2_-3b	3.26 m		2.93 m	
CH_2_- 4a	2.26 m	2.22 m	2.08 m	2.00 m
CH_2_-4b	1.99 m	1.95 m	1.92 m	1.85 m
CH_2_-5	1.92 m	2.04 m; 1.71 m	1.70 m	1.75 m

**Figure 7:**
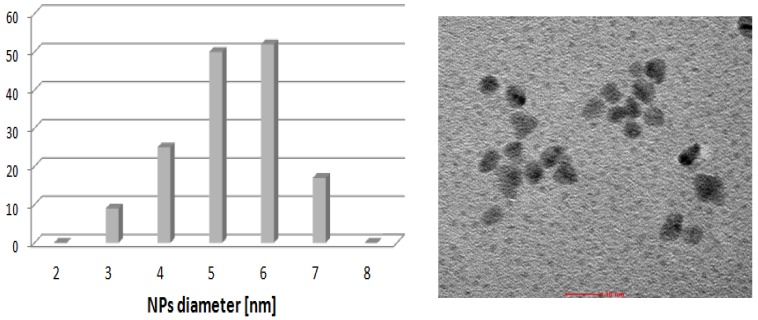
TEM micrograph and nanoparticles size distribution for the sample of [PdCl(allyl)]_2_ + [BA][L-PRO] heated in DMF (70 °C, 30 min).

The solution containing [BA][L-PRO] and [PdCl(allyl)]_2_ was heated at 70 °C for 30 min. During that time palladium was reduced and black powder was formed. Analysis of the solution by ^1^H-NMR shown only weak signals of L-prolinate anion while signals of allyl group were not detected. Thus, it was possible that palladium was eliminated from the solution as Pd(0) nanoparticles. To verify that hypothesis, TEM measurements were undertaken. Two analyses were performed, using water or methanol as solvent for the black palladium residue. In both samples Pd(0) nanoparticles were identified, partially agglomerated. Separated nanoparticles shown relatively narrow size distribution with maximum *ca.* 6 nm (Figure 7).

Pd(0) nanoparticles identified in the catalytic system most probably represent a resting state of the catalyst. In contact with reactants, in particular with aryl halide, solubilization of nanoparticles occurs with formation of catalytically active monomolecular species, similarly as it was proposed in other systems [30,31,33,39,41].

To estimate better the role of Pd(0) nanoparticles and underligated palladium species in the reaction course the Hg(0) test was performed using 500-fold excess of mercury to palladium. In the reaction performed in the presence of Hg(0) conversion was 32.6% while without Hg(0) 69.5% of substrate reacted. Thus, an inhibiting effect was observed, however soluble palladium species evidently participated in the reaction course.

**Figure 8:**
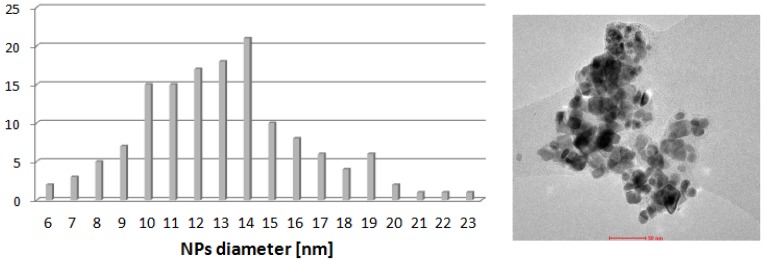
TEM micrograph and nanoparticles size distribution for the sample of [PdCl(allyl)]_2_ + [BA][L-PRO] heated in DMF in the presence of all components of the Heck reaction (70 °C, 30 min).

TEM analysis of the post-reaction mixture shown agglomeration of nanoparticles (Figure 8). The size of nanoparticles increased from *ca.* 6 nm to *ca.*, 13 nm and more regular shapes were observed. This observation confirms the conclusion that soluble forms of palladium participated in the catalytic reaction.

## 3. Experimental

### General Information

DHF, PhI, L-proline, [Bu_4_N]OH and [Bu_4_N][L-LACT] were obtained from Aldrich (Steinheim, Germany) and used without further purification. [BA][L-PRO] and [DDA][L-PRO] were obtained according to the literature [42]. [Bu_4_N][L-PRO] was obtained in reaction of [Bu_4_N]OH with L-proline. Pd(L-PRO)_2_ was obtained according to literature method [41].

### Heck Reaction

The Heck arylation of DHF with PhI was carried out under N_2_ atmosphere using standard Schlenk techniques. The reagents were introduced to the Schlenk tube (50 mL) in the following order: base (K_2_CO_3_ or NaOAc 4.34 mmol), palladium precursor (0.0356 mmol, 1% mol ), CIL (an appropriate weighed amount), solvent DMF (6 mL), PhI (0.4 mL, 3.57 mmol), DHF (0.7 mL, 8.59 mmol), mesitylene (internal standard, 0.15 mL). The reaction was carried out at 70 °C for 2 h. Afterwards, the reaction mixture was quenched with H_2_O (5 mL) and the organic products were separated by extraction with diethyl ether (3 times × 5 mL). The products were analyzed by GC-FID (Hewlett Packard 5890). Products **2**, **3**, **4** were identified by comparison of the MS spectra and the retention times with the literature data. The enantiomeric excess (ee) values were determined by GC-FID (Perkin Elmer Auto System XL) with a chiral β-cyclodextrin column.

## 4. Conclusions

We found an efficient catalytic system for arylation of DHF, composed of [PdCl(allyl)]_2_ and L-lactate or L-prolinate CILs. Addition of CIL to palladium precursor resulted in increase of iodobenzene conversion from 11% to 74.5%. When an effect of CIL is concerned, the highest conversion of iodobenzene was obtained with application of [Bu_4_N][L-LAC] for all studied palladium precursors.

It should be mentioned, that all ILs used in these studies were tetrabutylammonium salts which are known as very efficient stabilizing agents of nanoparticles [16,37,38,39]. Thus, the positive effect of ILs on the yield of the Heck reaction can be related to the stabilization of Pd(0) nanoparticles preventing their aggregation.

In all the reactions product **3** was obtained as the main one. The highest amount of product **3**, up to 59.2%, was formed in the reaction with PdCl_2_(PPh_3_)_2_ and [NBu_4_][L-LAC]. Unfortunately, the enantioselectivity was rather poor in that case, with ee values in the 4.3–5.5 range. Analysis of the enantioselectivity of the studied reactions allowed to indicate the best system, namely that composed of Pd_2_(dba)_3_ and [BA][L-PRO] in which the ee values for product **3** were in the range 10–13.3. The same CIL, [BA][L-PRO] generated **3** with ee 5.9–10 in the reaction with [PdCl(allyl)]_2_ and with ee equal 4.4–8.6 with PdCl_2_(cod). One can conclude that [BA][L-PRO] generates product **3** with the best although still unsatisfactory enantioselectivity.
